# High-Intensity Transient Signals Detected in a Renal Allograft

**DOI:** 10.1155/2023/9921063

**Published:** 2023-11-08

**Authors:** Lea Kaadi, Christele Lahoud, Samir Hachem, Tarek Smayra, Kamal Hachem

**Affiliations:** ^1^Medical Imaging Department, Hôtel-Dieu de France, Alfred Naccache Boulevard, Achrafieh, Beirut, Lebanon; ^2^Faculty of Medicine, University of Saint Joseph, Beirut, Lebanon

## Abstract

High-intensity transient signals (HITS) are signals recorded by the Doppler ultrasounds, reflecting either the passage of microemboli, both solid or gaseous in the vessels, or artifacts. Their identification during Duplex US highlights the need for further evaluation to rule out a potential embolic source. A 49-year-old female was referred to our hospital for renal transplantation. The Doppler ultrasound done on day 4 after the surgery revealed the presence of high-intensity transient signals (HITS) suggesting the passage of an emboli. Renal magnetic resonance angiography (MRA) confirmed the presence of peripheral parenchymal defects suggestive of a distal embolus. A better understanding and recognition of this radiological sign are essential in order to initiate appropriate patient management when needed. In this report, we review the importance of HITS and present a case in which HITS were detected in an unusual location: an allograft kidney artery.

## 1. Introduction

Despite advances in postrenal transplant patient care, the prevalence of renal graft losses due to vascular complications (i.e., renal artery or vein stenosis/thrombosis, renal infarct, pseudoaneurysm, and arteriovenous fistula…) remains high [1, 2]. This emphasizes the need for effective renal allograft surveillance to reduce the risk of graft dysfunction and promote its survival. Imaging, specifically renal Doppler ultrasound, plays a crucial role in the early detection of potential vascular complications of the allograft [3–6].

HITS is a valuable ultrasonographic sign used to detect microemboli, relying on ultrasound waves' backscattering in the vessel, resulting in embolic and high-intensity transient signals [7]. The recognition of this US finding is mostly limited to transcranial and carotid Doppler ultrasounds [7, 8]. To our knowledge, only a few cases of HITS have been reported in peripheral organs, and outside the settings of a transcranial Doppler ultrasound [9]. This case reports the finding of a transient embolic signal or HITS in a renal allograft's artery during a posttransplant surveillance Doppler ultrasound.

## 2. Case Presentation

We report the case of a 49-year-old female smoker with hypertension and end-stage renal disease, admitted to undergo a renal transplant on December 22, 2022. The patient's chronic kidney failure was diagnosed at a late stage, and she has been undergoing hemodialysis since 2013. She had no past surgical, psychosocial, or family history. The patient was asymptomatic at the time of her admission.

The patient had a baseline creatinine level of around 5.7 mg/dL, with a glomerular filtration rate of less than 10. A recent cardiac ultrasound was performed in April 2022. No intracardiac thrombus was seen.

The patient was considered eligible for surgery, and the renal transplant, which involved a living donor, was performed without complications.

Warm ischemia time was 2 minutes, cold ischemia time was 1 hour and 13 minutes, and time of revascularization was 1 hour.

Immunosuppressive medications were initiated postsurgery. The patient remained stable postoperatively, with improving blood examinations, including a serum creatinine level of 0.84 mg/dL four days after surgery (an 85.3% reduction from baseline), and satisfactory diuresis.

**Figure 1:**
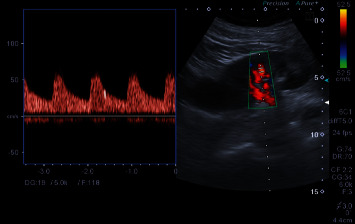
Spectral Doppler records HITS in a distal arterial branch of the allograft kidney. Embolic signal on renal Doppler recording of the distal arterial branch of the allograft kidney. The arrow indicates the high-intensity transient signals (HITS) seen suggesting the detection of a microemboli.

On day 4 after surgery, a follow-up Doppler renal ultrasound was performed. The renal graft, measuring 10.5 cm and localized in the right iliac fossa, appeared well differentiated. The renal cavities were not dilated, and the graft appeared well-vascularized with normal intrarenal vascular perfusion in both the renal artery and vein. Blood flow inside the graft showed a satisfying systolic upstroke, with a normal resistance index of 0.61. However, an interesting finding during the Doppler ultrasound was the recording of “high-intensity transient signals” (HITS) suggestive of emboli (Figure 1.). When repeating the Doppler exam within few minutes, no other signals were recorded.

Clinically, the patient remained stable, with no symptoms and no particular abnormalities except for a high lactate dehydrogenase (534 U/L) and an occasional high blood pressure readings, which were managed with a calcium channel blocker was initiated.

**Figure 2:**
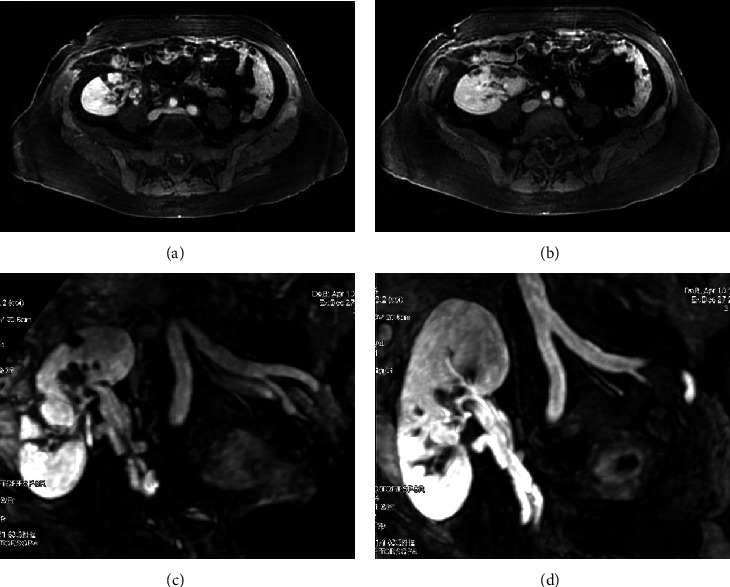
Magnetic resonance angiography (MRA) reconstructed MIP imaging showing peripheral parenchymal defects (a, b) and the permeability of the artery and the vein's graft (c, d).

To further assess the findings of the renal ultrasound, a renal magnetic resonance angiography (MRA) was performed the following day, revealing peripheral parenchymal defects limited to the distal part of the anterior aspect of the graft, suggestive of a distal embolus (Figure 2).

Although a significant reduction in the diameter of the external iliac artery was noted, adjacent metallic clips may have contributed to a false appearance of stenosis due to magnetic interference.

Apart from those defects, no visible stenosis or thrombi were found in the first divisions of the graft's artery which were of normal appearance. No other stenosis or thrombi were found in the graft's artery or vein. The graft vein appeared permeable, and no dilatation of the uretero-pyelocaliceal cavities of the graft was noted.

The patient was closely monitored, and laboratory blood work and clinical assessment continued to improve (i.e., creatinine value of 0.79 mg/d; hemoglobin and hematocrit level of 9.5 g/dL and 29.8%, respectively).

Given the imaging findings and the biological laboratory findings, an oral anticoagulant was prescribed for 6 months. The patient was discharged on day 9 postsurgery for outpatient follow-up.

## 3. Discussion

High-intensity transient signals (HITS) or microembolic signals (MES) are recorded by the Doppler ultrasound, reflecting the passage of microemboli, both solid and gaseous, in the vessel, or they can be artifacts [10]. The Doppler ultrasound measures blood velocity, which depends on the frequency difference between transmitted and reflected ultrasound waves, determined by the acoustic impedance and size of the blood. Emboli create higher-intensity signals compared to signals reflected by erythrocytes, seen during diastole, systole, or both [11]. MES are characterized by being transient (lasting less than 300 msec), unidirectional high intensity (intensity greater than 3 db), and producing a sound called “chirp snap” [12]. The embolic source can be arterial (from an atherosclerotic plaque, or parietal thrombus), cardiac (from a mechanical valve or a valvular pathology), or venous (in the case of a paradoxical embolism on a permeable foramen ovale) [13].

The first Doppler detections of HITS were made in the 1960s during an open-heart surgery [14].

To our knowledge, there are no reported cases of HITS in transplant renal arteries. Most reports of embolic signals or HITS are related to cerebral arteries during transcranial Doppler ultrasound, especially in patients at high risk for cardioembolic strokes (i.e., atrial fibrillation, carotid stenosis, mechanical heart valves…) [7, 15], coagulopathies [16], or cardiothoracic procedures, such as LVAD or heart defect repair surgeries requiring a cardiopulmonary bypass [8, 17]. However, only a few cases of HITS have been reported during Duplex US in organs other than the brain.

Indeed, in a case report by Dimitrov et al. [18], they described the recording of HITS in the outflow graft of an LVAD during a transthoracic US. Woltmann et al. [9] noted MES during a hemodialysis session in two patients, one with a synthetic graft and the other with an arteriovenous fistula. These signals could be due, among other possible causes, to the passage of microemboli in the circulation [9].

Given the long-term and repetitive monitoring needed to evaluate the function and vitality of the renal allograft posttransplantation, Duplex renal ultrasound is the modality of choice and the established method for allograft surveillance. This is due to the nonionizing, noninvasive nature of this tool and the accessible location of renal grafts in the iliac fossa [19, 20].

In the presented case, HITS were recorded on the Doppler ultrasound while assessing the structure and the vasculature of the transplanted kidney.

The finding of HITS during the conduction of the Doppler US using the Toshiba Aplio 500 using 6C1 Convex Probe 1-6 MHz on the transplant renal artery could suggest the passage of microemboli through the vessel. HITS have been shown to indicate the migration of solid or gaseous emboli through the vessel when perceived during transcranial Doppler [21, 22]. The clinical significance of high-intensity transient signals is not fully understood [12, 21], and further studies are needed to evaluate their clinical value in renal allograft arteries and their implications in renal Duplex US. It is important to note that while HITS are commonly associated with microemboli detection, other causes such as artifacts and flow disturbances cannot be excluded [12].

Signals that are both above and below the baseline, or that are bidirectional, frequently signify artifacts [23]. In our case, a unidirectional signal within the flow spectrum was detected.

An angio-MRI was performed the following day using a GE Signa Excite HDx 3.0T 2006 equipped with enhanced gradients/Echospeed, using a Torso coil.

The imaging method was chosen for this case, because unlike the angio CT scanner, it does not require iodinated contrast, thus avoiding the risk of renal impairment in a newly transplanted patient.

The MRI showed peripheral parenchymal defects, limited to the distal part of the anterior aspect of the graft. This finding could be explained by the emboli detected with the Doppler ultrasound.

It has been commonly reported that cold ischemia time influences kidney allograft survival and function [24], while prolonged warm ischemia time affects the hospital stay and the long-term graft survival [25]. But no complications were reported during the renal transplant which was underwent, nor during the short cold and warm ischemia time. To our knowledge, no studies were conducted to assess the correlation between prolonged cold and/or warm ischemia time with radiologic features.

Many early postoperative complications can occur after renal transplantation. They are categorized in a timely manner depending on the timing at which they took place following the transplantation: early or late [3, 4].

These complications can include issues related to surgical technique (i.e., hemorrhage, pseudoaneurysm, and arteriovenous fistula), renal allograft compartment syndrome, and vascular complications such as thrombosis of the transplant renal vein or artery [26].

In the presented case, no arterial or venous thrombosis was detected in the Doppler ultrasound or the angio-MR. Moreover, the imaging did not show any pseudoaneurysm nor an arteriovenous fistula.

In addition, focal parenchymal lesions in renal transplants can have many causes including pyelonephritis, segmental infarction, parenchymal neoplastic mass, and allograft cysts [19]. The patient had no symptoms suggesting a renal infection. The focal parenchymal lesions did not enhance after gadolinium injection and did not show a liquid signal, ruling out the possibility of a parenchymal neoplastic mass and allograft cysts, respectively. This confirmed that it was a segmental infarction due to an embolus.

The patient did not have valvular problems or a mechanical valve but presented several aortic atherosclerotic plaques, which could support the previously described finding.

The highly operator-dependent nature of duplex ultrasound and the transient nature of embolic signals constitute limitations in the detection of HITS. Moreover, the clinical significance of HITS is still not fully understood. While the implications following the identification of embolic signals in renal allograft arteries remain disputable, their detection could be of great value in taking precocious measures to ensure the survival and vitality of the renal graft and preventing its loss of function. Another limitation is the uncertainty as to the origin of HITS, which can sometimes be due to artifacts or flow disturbances. Specific consensus has already been established for the characterization of MES in order to reduce erroneous identifications and guide their recognition by physicians [22].

## 4. Conclusion

Although high-intensity transient signals are not commonly recorded on spectral Doppler, radiologists should recognize and interpret them to improve patient management. Further research is needed to understand the clinical and therapeutic implications of embolic signals and HITS in ultrasonographic monitoring of renal allografts, as well as their role in predicting ischemic events and graft outcomes.
